# Melatonin protects TEGDMA-induced preodontoblast mitochondrial apoptosis via the JNK/MAPK signaling pathway: Melatonin protects TEGDMA-induced preodontoblast mitochondrial apoptosis

**DOI:** 10.3724/abbs.2023263

**Published:** 2024-02-01

**Authors:** Qihao Yu, Ruize Hua, Bingyang Zhao, Dongchao Qiu, Chengfei Zhang, Shengbin Huang, Yihuai Pan

**Affiliations:** 1 Department of Endodontics School and Hospital of Stomatology Wenzhou Medical University Wenzhou 325000 China; 2 Institute of Stomatology School and Hospital of Stomatology Wenzhou Medical University Wenzhou 325000 China; 3 Restorative Dental Sciences Endodontics Faculty of Dentistry The University of Hong Kong Hong Kong SAR 999077 China; 4 Department of Prosthodontics School and Hospital of Stomatology Wenzhou Medical University Wenzhou 325000 China

**Keywords:** TEGDMA, apoptosis, mitochondrial dysfunction, dental pulp cell, melatonin, MAPK

## Abstract

Resin monomer-induced dental pulp injury presents a pathology related to mitochondrial dysfunction. Melatonin has been regarded as a strong mitochondrial protective bioactive compound from the pineal gland. However, it remains unknown whether melatonin can prevent dental pulp from resin monomer-induced injury. The aim of this study is to investigate the effects of melatonin on apoptosis of mouse preodontoblast cells (mDPC6T) induced by triethylene glycol dimethacrylate (TEGDMA), a major component in dental resin, and to determine whether the JNK/MAPK signaling pathway mediates the protective effect of melatonin. A well-established TEGDMA-induced mDPC6T apoptosis model is adopted to investigate the preventive function of melatonin by detecting cell viability, apoptosis rate, expressions of apoptosis-related proteins, mitochondrial ROS (mtROS) production, mitochondrial membrane potential (MMP) and adenosine triphosphate (ATP) level. Inhibitors of MAPKs are used to explore which pathway is involved in TEGDMA-induced apoptosis. Finally, the role of the JNK/MAPK pathway is verified using JNK agonists and antagonists. Our results show that melatonin attenuates TEGDMA-induced mDPC6T apoptosis by reducing mtROS production and rescuing MMP and ATP levels. Furthermore, mitochondrial dysfunction and apoptosis are alleviated only by the JNK/MAPK inhibitor SP600125 but not by other MAPK inhibitors. Additionally, melatonin downregulates the expression of phosphorylated JNK and counteractes the activating effects of anisomycin on the JNK/MAPK pathway, mimicking the effects of SP600125. Our findings demonstrate that melatonin protects mDPC6T cells against TEGDMA-induced apoptosis partly through JNK/MAPK and the maintenance of mitochondrial function, offering a novel therapeutic strategy for the prevention of resin monomer-induced dental pulp injury.

## Introduction

Due to the superior performance, ease of operation and aesthetic properties, resin-containing compounds are being used for a wide variety of dental applications, such as restorations, sealants, bonding agents, and dental pulp capping [ 1‒ 3]. The most commonly used monomer compounds in dental resins are bisphenol-A-glycidyl methacrylate (Bis-GMA), 2-hydroxyethyl methacrylate (HEMA) and triethylene glycol dimethacrylate (TEGDMA) [4]. Depending on the application, different amounts of the resin compounds mentioned above depolymerize and release residual monomers into oral tissues [5]. Depolymerized resin monomers can diffuse across the dentin layer through the dentinal tubules, with concentrations ranging from 0.2 to 8 mM [ 6, 7]. This diffusion can trigger a wide variety of cellular responses in the pulp tissue, including apoptosis, inflammation, and impairment of dental mineralization. Apoptosis is the primary manifestation of resin monomer-induced dental pulp injury, commonly observed as a result of cell toxicity [ 8‒ 11], which not only aggravates the existing damage but also obstructs the defense responses of the dentin-pulp complex. Therefore, exploring the mechanisms of TEGDMA-induced cell apoptosis and finding potential preventive/therapeutic strategies are essential for improving restorative materials for clinical application.

Mitochondria play vital roles in many cellular processes, including cell proliferation, metabolism, and apoptosis [ 12‒ 14]. Accumulating evidence has indicated that an excess of reactive oxygen species (ROS) in mitochondria promotes caspase-dependent apoptosis. As a result, mitochondria are identified as the center of apoptosis through the intrinsic pathway [15]. Furthermore, an excess of ROS causes respiratory dysfunction and reduces adenosine triphosphate (ATP) generation, which further promotes mitochondrial ROS (mtROS) production and oxidative damage, thereby creating a vicious cycle. Previous research has shown that the cytotoxicity of TEGDMA to oral tissues leads to an accumulation of mtROS and irreversible mitochondrial damage [ 16, 17]. Our latest study also demonstrated that mitochondrial dysfunction is the major factor in preodontoblast apoptosis induced by TEGDMA [18]. However, the detailed mechanism, especially the signaling pathway that regulates mitochondrial dysfunction in TEGDMA-induced cell apoptosis, needs further exploration.

The mitogen-activated protein kinase (MAPK) pathway, grouped into distinct families, such as extracellular signal-regulated protein kinase 1/2 (ERK1/2), c-Jun N-terminal kinase (JNK), and p38 MAPK, has been proven to be essential for regulating various cellular processes, including cell proliferation, differentiation, and apoptosis [ 19, 20]. The role of the MAPK pathway in mitochondria focuses on the phosphorylation of executive function proteins that are essential for fundamental mitochondrial functions, such as energy production, redox processes, and metabolic pathways [21]. Meanwhile, the subfamilies of MAPKs have distinct functions within the mitochondria. JNK has been implicated in the generation of high levels of mtROS when it is transferred to mitochondria [22], while ERK1/2 and p38 act as upstream signals leading to disruptions in mitochondrial membrane potential, cytochrome C release, and caspase3 activation [23]. On the other hand, in resin monomer-induced mouse macrophage apoptosis, the activation of ERK/JNK/p38 MAPKs induced by TEGDMA is inhibited by the antioxidant N-acetylcysteine (NAC) [24]. Another study noted that p38 and ERK1/2, but not JNK, participated in TEGDMA-induced cell apoptosis [25]. Therefore, further clarification is needed regarding which MAPK pathway is involved in TEGDMA-induced apoptosis in preodontoblasts and the identification of a mitochondrion-targeted antioxidant capable of effectively sequestering mtROS and protecting against mitochondrial damage by this mechanism.

Melatonin is synthesized and secreted mainly by the pineal gland during the alternation of light and darkness. Similarly, melatonin is commonly ingested in the form of drugs, serving as an oral mitochondrial protective agent [26]. Melatonin has been employed in the fields of oral diseases and material applications. For instance, it has been shown to alleviate the symptoms of dental pulpitis [27] and promote the osteointegration of dental implants [28]. Furthermore, as an indoleamine, melatonin may offer a new potential application to enhance the properties of biomaterials used in dentistry [29]. Serving as a potent mitochondrial protective substance, melatonin enhances mitochondrial electron transfer chain (ETC) complexes I and IV, thereby improving mitochondrial respiration, ATP synthesis, and energy metabolism under stress conditions [30]. Evidence suggests that melatonin attenuates apoptosis induced by hydrogen peroxide in human dental pulp cells (hDPCs) [ 31, 32]. Physiological concentrations of melatonin can inhibit proliferation and promote odontogenic differentiation of hDPCs [ 33, 34]. It has been reported that exogenous melatonin can ameliorate oxidative stress damage mediated by the MAPK pathway [35], but its specific effect on dental pulp cell apoptosis and mitochondrial damage induced by TEGDMA remains unknown.

In this study, we utilized an established *in vitro* model to investigate the impact of melatonin on TEGDMA-induced mitochondrial dysfunction and apoptosis in preodontoblasts. Our aim was also to elucidate the role of the MAPK signaling pathway in the protective effect of melatonin. This study represents the first exploration of the protective effects of the bioactive compound melatonin on TEGDMA-induced dental pulp damage, shedding light on its role through the MAPK signaling pathway, marking an innovative approach in this field.

## Methods and Materials

### Reagents

Cell culture medium and supplements, MitoSOX Red (#M36008), MitoTracker Green (Mitogreen) (#M7514), and tetramethylrhodamine methyl ester (TMRM) (#T668) were purchased from Life Technologies (Grand Island, USA). Triethylene glycol dimethacrylate (TEGDMA) (#261548), 3-(4,5-dimeth-ylthiazol-2-yl)-2,5-diphenyltetrazolium bromide (MTT) (#M2128), melatonin (#M5250), SP600125 (#S5567), PD98059 (#P215) and SB203580 (#S8307) were obtained from Sigma-Aldrich (St Louis, USA). Antibodies against phosphorylated JNK (Thr183/Tyr185) (p-JNK) (#4668S), JNK (#9252S), phosphorylated ERK1/2 (Thr202/Tyr204) (p-ERK1/2) (#4370S), ERK1/2 (#4969S), phosphorylated p38 (Thr180/Tyr182) (p-p38) (#4511S), p38 (#8690S), Bax (#2772S), caspase3 (#9662S) and β-actin (#3700) were obtained from Cell Signaling Technology (Beverly, USA). The annexin V-fluorescein isothiocyanate (FITC) apoptosis detection kit, chamber slides, and goat anti-rabbit (#656120) and anti-mouse (#626520) secondary antibodies were obtained from Invitrogen (Carlsbad, USA). ATP assay kit (#S0026) was obtained from Beyotime (Shanghai, China). Anisomycin (#S7409) was obtained from Selleckchem (Shanghai, China). Mitoquinone (MitoQ) (#89950) was obtained from Cayman Chemical (Ann Arbor, USA).

### Cell culture

mDPC6T cells, a preodontoblast cell line, were generously provided by Prof Chen from Wuhan University (Wuhan, China). Cells were maintained in Dulbecco’s modified Eagle’s medium (DMEM) supplemented with 10% fetal bovine serum and antibiotics (100 IU/mL penicillin G and 100 ng/mL streptomycin) in a humidified incubator at 37°C with 5% CO _2_.

### Cell treatments

The test compounds were prepared as stock solutions and diluted to the desired final concentrations immediately before use. Apoptosis was induced in mDPC6T cells by treatment with TEGDMA (2 mM) for 6 h [ 18, 36]. To examine the protective effect of melatonin on TEGDMA-induced mDPC6T damage, varying concentrations of melatonin (50, 100, 150, and 200 μM) were added along with TEGDMA to the culture medium. The final concentrations of the other compounds used were as follows: melatonin (100 μM), MitoQ (10 nM), SP600125 (10 μM), PD98059 (10 μM), SB203580 (10 μM), and anisomycin (1 μM). The cells were pretreated with the above mentioned drugs for 2 h and then exposed to TEGDMA. The final concentration of dimethyl sulfoxide (DMSO) in the culture did not exceed 0.5% in the experiments. Cells were subjected to the designated treatments with or without TEGDMA and the specified test compounds for various durations, following the experimental protocol.

### Cell viability test

mDPC6T cells were seeded in 96-well plates (1×10 ^4^ cells/well) and cultured under different conditions, as indicated for each experiment. mDPC6T cells were briefly washed twice with phosphate buffered saline (PBS) and incubated in 100 μL/well serum-free medium supplemented with 20 μL MTT solution (5 mg/mL) at 37°C. After 6 h of incubation, the supernatant was removed, and the formazan crystals were dissolved in 100 μL/well of DMSO for 10 min. Then, the plates were agitated for 15 s, and the absorbance was measured at 570 nm using a microplate reader (Molecular Devices, San Jose, USA).

### Apoptosis measurement by flow cytometry

Flow cytometry was performed to identify the cell cycle and apoptotic cells. Annexin-V labelled with fluorescein isothiocyanate and propidium iodide (PI, 1 μg/mL) was used to determine cell apoptosis and necrosis. After exposure to various experimental conditions, cells were trypsinized and labelled with fluorochromes at 37°C, and then cytofluorometric analysis was performed with a FACScan flow cytometer (Becton Dickinson, Franklin Lakes, USA).

### Western blot analysis

After the indicated treatments, cells were collected and lysed in cell lysis buffer (Cell Signaling Technology). Protein concentrations were determined using a Bradford protein assay kit (Thermo Fisher Scientific, Waltham, USA). Proteins were separated by SDS-PAGE and transferred to a polyvinylidene difluoride (PVDF) membrane (Millipore, Billerica, USA). Anti-p-JNK (1:2000), anti-JNK (1:2000), anti-ERK1/2 (1:2000), anti-p-ERK1/2 (1:2000), anti-p-p38 (1:2000), anti-p38 (1:2000), anti-Bax (1:2000), anti-caspase3 (1:1000) and anti-β-actin (1:8000) antibodies were used as primary antibodies to incubated with the membrane. After incubation with an appropriate HRP-conjugated secondary antibody (1:4000), protein bands were visualized using an enhanced chemiluminescence (ECL) substrate (Thermo Fisher Scientific), detected using a Bio-Rad imaging system (Bio-Rad, Hercules, USA), and quantified with ImageJ software (NIH, Bethesda USA).

### Mitochondrial functional imaging assay

Cells were seeded in chamber slides at 1×10 ^4^ cells/well and then treated with TEGDMA and other test compounds for 1 h. Then, the cells were incubated in fresh culture medium containing 2 μM MitoSOX for 30 min. To assess the MMP, cells were costained with Mitogreen (100 nM) and TMRM (100 nM) for 30 min, according to our previous work [18], and images were captured under a fluorescence microscope (ZEISS, Jena, Germany). Excitation wavelengths were 543 nm for MitoSOX and TMRM and 488 nm for Mitogreen. Postacquisition processing was performed with ImageJ software (NIH) to measure and quantify fluorescence signals. Mitochondrial fluorescence intensities, density, and length were quantified by an investigator blinded to the experimental groups. More than 30 clearly identifiable mitochondria in 10 to 15 randomly selected cells per experiment were measured according to our previous work [18].

### ATP detection

To measure ATP level, whole-cell extracts from the indicated cells were lysed in lysis buffer provided in the ATP Assay kit (Beyotime). After centrifugation at 12,000 *g* for 5 min at 4°C, the supernatants were transferred to a new 1.5-mL tube for ATP testing. The luminescence from a 100 μL sample was assayed in a luminometer (Molecular Devices, San Jose, USA) together with 100 μL of ATP detection buffer. The standard curve of ATP concentration was prepared from a known amount (1 nM to 1 μM). All measurements were performed in triplicate.

### Statistical analysis

Data are presented as the mean±SD. Statistical analysis was performed using StatView software (Version 5.0.1; SAS Institute, Cary, USA). For comparisons between multiple groups, one-way ANOVA was used followed by individual post hoc Fisher tests when applicable. *P*<0.05 was considered statistically significant.

## Results

### Melatonin attenuates TEGDMA-induced apoptosis and mitochondrial dysfunction in mDPC6T cells

**Figure 1:**
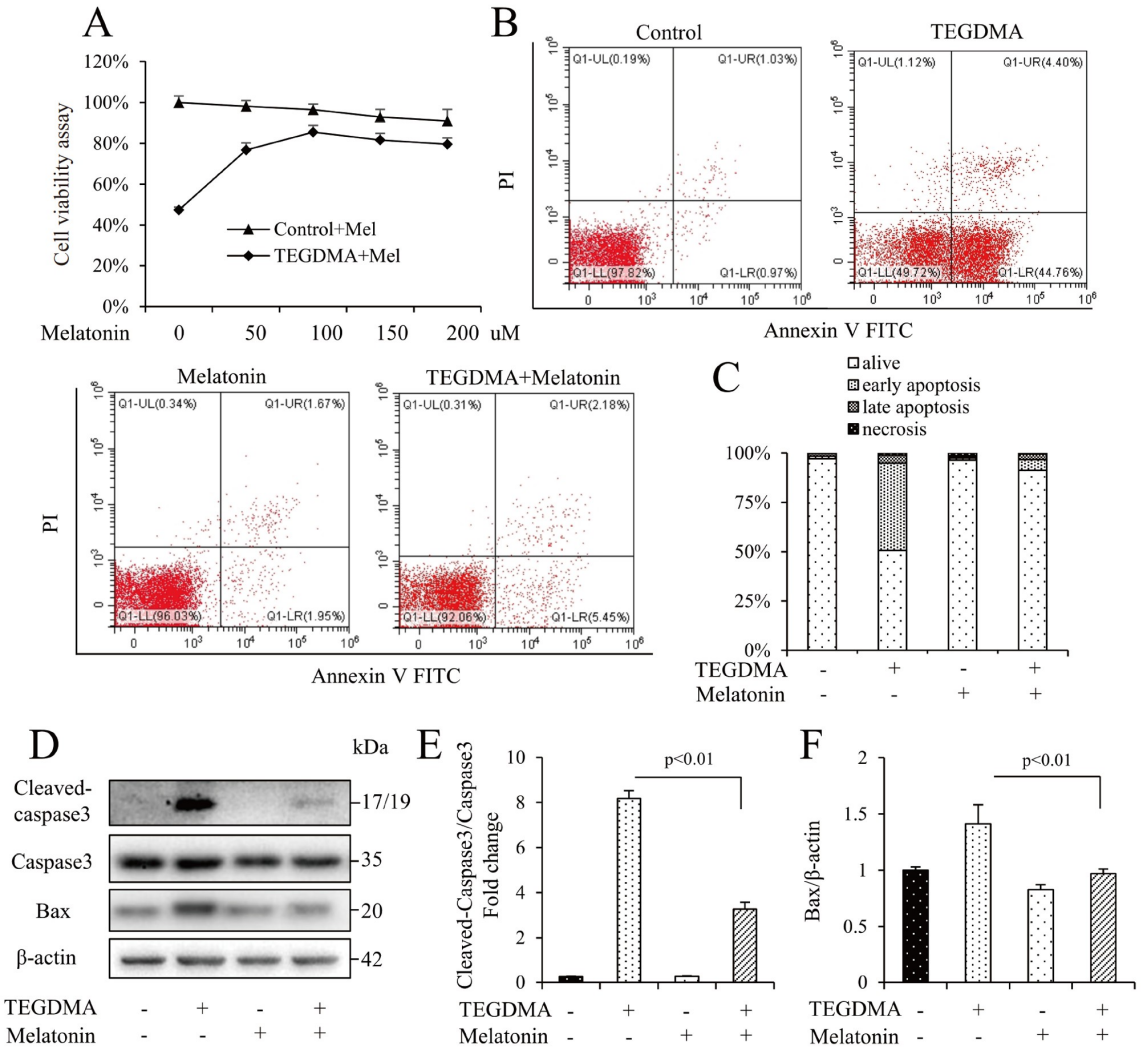
Figure 1 Melatonin attenuated TEGDMA-induced apoptosis in mDPC6T cells (A) Cell viability determined by MTT in mDPC6T cells in the presence of melatonin (Mel) with or without TEGDMA. Data are presented as the mean±SD (n=3). (B,C) Flow cytometry assay after melatonin treatment. Data are presented as the mean±SD (n=3). (D) Representative western blots for caspase3 and Bax in mDPC6T cells with (+) or without (‒) melatonin treatment in the presence of TEGDMA (+) or culture medium (‒). (E) Quantification of protein expression of cleaved caspase3 relative to caspase3. (F) Quantification of protein expression of Bax relative to β-actin. Data are presented as the mean±SD (n=3).

**Figure 2:**
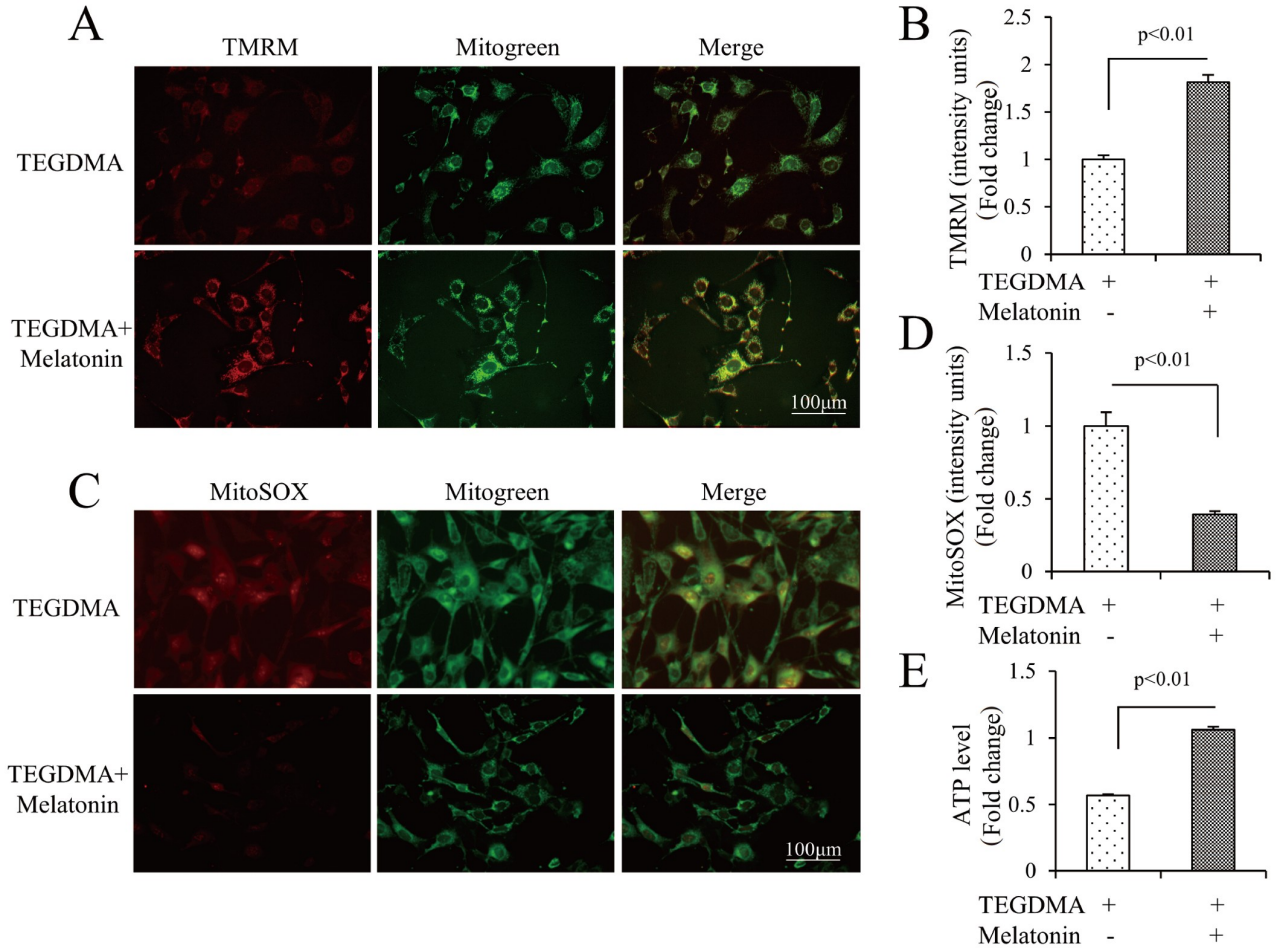
Figure 2 Melatonin ameliorated TEGDMA-induced mitochondrial dysfunction in mDPC6T cells (A,B) Representative images showing TMRM staining (A) and quantification (B) in the indicated groups (n=3). (C,D) Representative images of MitoSOX staining (C) and quantification (D) in the indicated groups (n=3). (E) Cellular ATP levels in the indicated groups (n=3).

As we previously discovered, TEGDMA reduced cell viability and induced apoptosis partially due to mitochondrial dysfunction in mDPC6T cells. Based on previous studies, we utilized mDPC6T cells treated with 2 mM TEGDMA for 6 h as our experimental group [ 18, 36 ]. Melatonin, an active circadian regulator secreted by the pituitary gland and distributes throughout the body [26], including oral tissues, has been shown to have strong mitochondria-targeted antioxidant and antiapoptotic effects on various cell types. To explore the effect of melatonin on TEGDMA-induced mDPC6T cell apoptosis, we treated mDPC6T cells with melatonin 2 h prior to TEGDMA exposure. As shown in Figure 1A, melatonin partially rescued the viability of mDPC6T cells. Moreover, the DNA damage and apoptosis induced by TEGDMA were significantly ameliorated by melatonin, as reflected by flow cytometry ( Figure 1B,C). Melatonin also downregulated the expression levels of Bax and cleaved caspase3 induced by TEGDMA ( Figure 1D‒F). These data indicated that melatonin attenuated TEGDMA-induced mDPC6T cell apoptosis. It is clear that TEGDMA-induced mDPC6T mitochondrial reactive oxygen species elevation and membrane potential decrease were significant [ 18, 36]. Interestingly, melatonin significantly ameliorated mitochondrial ROS levels, as indicated by reduced MitoSOX staining intensity ( Figure 2C,D). Compared with TEGDMA alone, melatonin improved MMP level, as shown by increased TMRM intensity ( Figure 2A,B). In addition, we evaluated the effects of melatonin on mitochondrial energy-producing function. Compared with TEGDMA alone, melatonin rescued the cellular ATP level ( Figure 2E). In summary, melatonin ameliorated TEGDMA-induced apoptosis and mitochondrial dysfunction in mDPC6T cells.

### JNK/MAPK pathway is involved in TEGDMA-induced mDPC6T mitochondrial apoptosis

**Figure 3:**
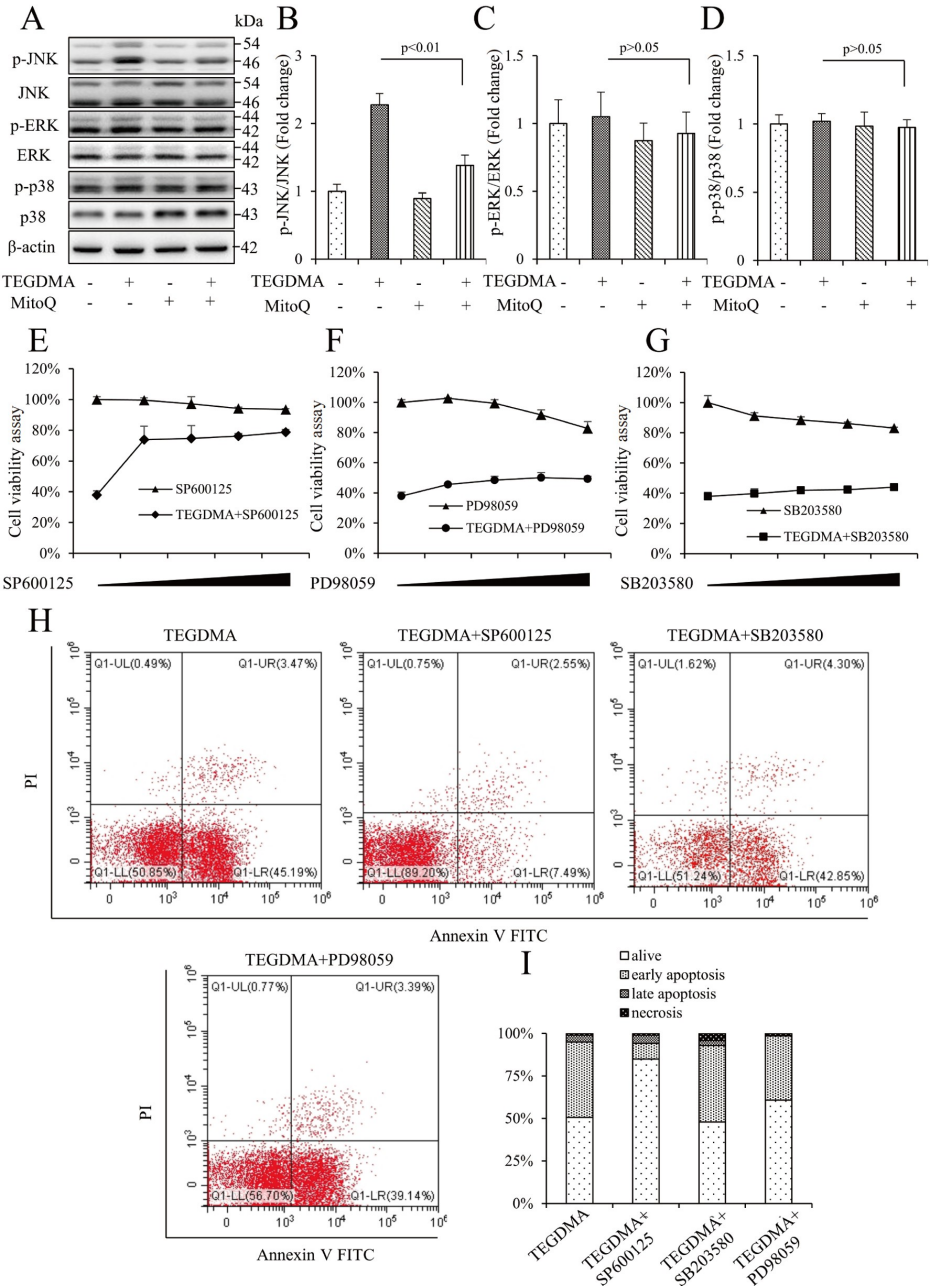
Figure 3 The role of the MAPK pathway in TEGDMA-induced mDPC6T cell apoptosis (A) Representative western blots for MAPKs in mDPC6T cells with (+) or without (‒) MitoQ treatment in the presence of TEGDMA (+) or culture medium (‒). (B) Quantification of protein expression of p-JNK relative to JNK. (C) Quantification of protein expression of p-ERK relative to ERK. (D) Quantification of protein expression of p-p38 relative to p38. Data are presented as the mean±SD (n=3). (E‒G) Cell viability determined by MTT after TEGDMA with or without treatment with MAPK inhibitors. Data are presented as the mean±SD (n=3). (H,I) Flow cytometric analysis after TEGDMA with or without treatment with MAPK inhibitors. Data are presented as the mean±SD (n=3).

**Figure 4:**
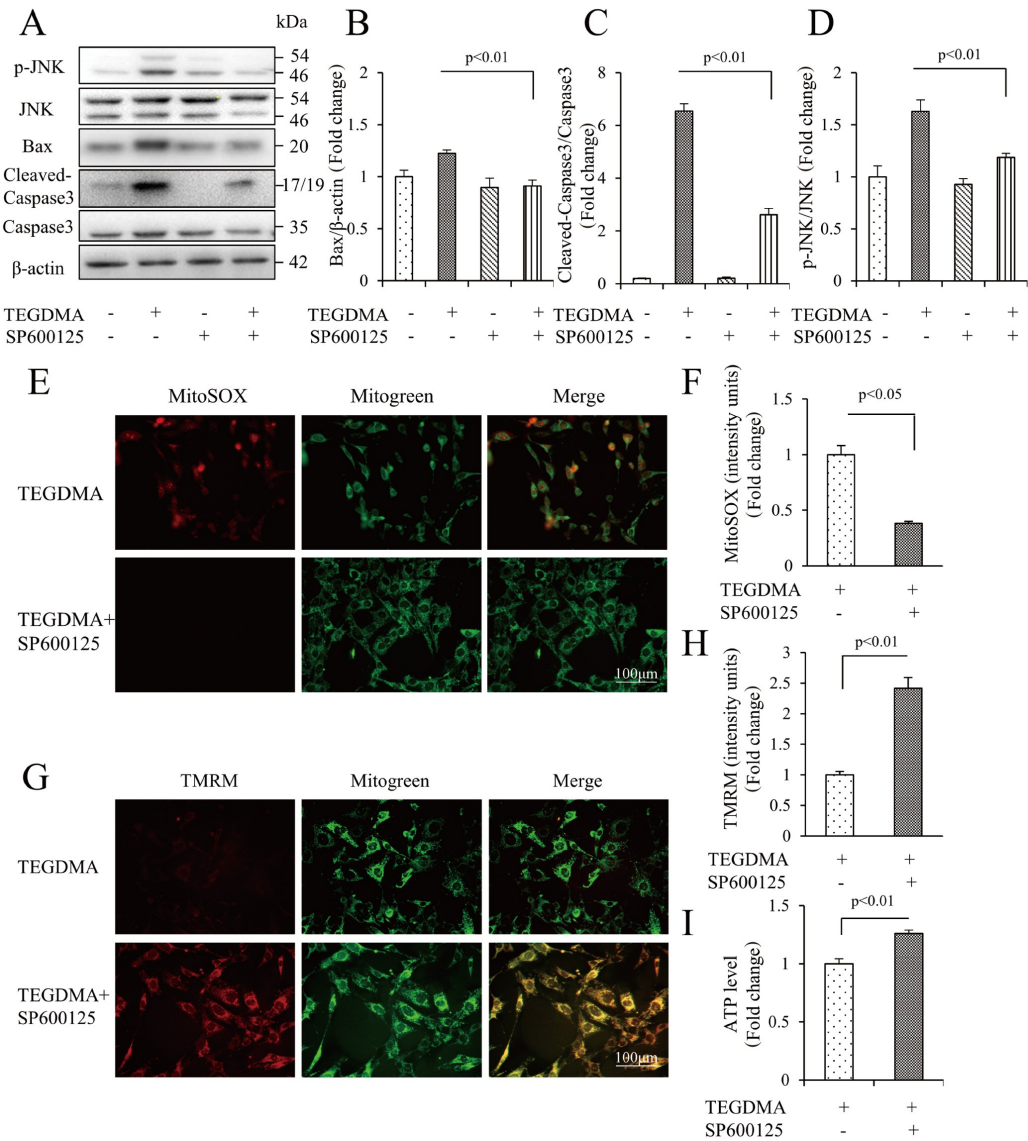
Figure 4 The role of the JNK/MAPK pathway in TEGDMA-induced mDPC6T apoptosis and mitochondrial dysfunction (A) Representative western blots in mDPC6T cells with (+) or without (‒) SP600125 treatment in the presence of TEGDMA (+) or culture medium (‒). (B) Quantification of protein expression of Bax relative to β-actin. (C) Quantification of protein expression of cleaved caspase3 relative to caspase3. (D) Quantification of protein expression of p-JNK relative to JNK. Data are presented as the mean±SD (n=3). (E,F) Representative images showing MitoSOX staining (E) and quantification (F) in the indicated groups (n=3). (G,H) Representative images of TMRM staining (G) and quantification (H) in the indicated groups (n=3). (I) Cellular ATP levels in the indicated groups (n=3).

We then examined whether TEGDMA-induced mDPC6T apoptosis and mitochondrial dysfunction involve MAPK family proteins, including p38, JNK, and ERK1/2. We utilized MitoQ, a classical mitochondrial function protectant, to investigate the role of the MAPK pathways in the model. The results revealed that TEGDMA stimulation of mDPC6T cells could enhance the phosphorylation level of JNK but not that of p38 or ERK1/2 ( Figure 3A‒D). In addition, pretreatment with MitoQ reduced the phosphorylation level of JNK without affecting the other MAPK pathways ( Figure 3A‒D). To further confirm the role of the MAPK signaling pathway, we used chemical inhibitors of MAPKs, namely, SP600125, PD98059, and SB203580, which specifically inhibit the actions of JNK, ERK1/2, and p38 respectively, to evaluate their ability to rescue TEGDMA-induced mDPC6T cell apoptosis. MTT assays demonstrated that only SP600125 protected against the cytotoxic effect of TEGDMA on mDPC6T cells ( Figure 3E‒G). Simultaneously, flow cytometric analysis results revealed the reversal effect of SP600125 on apoptotic cells, whereas the reversal effects of PD98059 and SB203580 were less apparent ( Figure 3H,I). Furthermore, western blot analysis results indicated that SP600125 reduced the protein expressions of cleaved caspase3 and Bax ( Figure 4A‒D), indicating that SP600125 safeguarded mDPC6T cells from TEGDMA-induced apoptosis. Subsequently, we investigated the role of JNK/MAPK signaling in TEGDMA-induced mitochondrial dysfunction. Our findings suggested that SP600125 ameliorated mDPC6T mitochondrial dysfunction, as evidenced by the reduction in mtROS production ( Figure 4E,F), elevation in MMP ( Figure 4G,H), and cellular ATP level ( Figure 4I). In summary, these results provide direct evidence that the JNK/MAPK pathway significantly participates in TEGDMA-induced mDPC6T mitochondrial apoptosis.

### Melatonin prevents TEGDMA-induced apoptosis through the JNK/MAPK pathway

**Figure 5:**
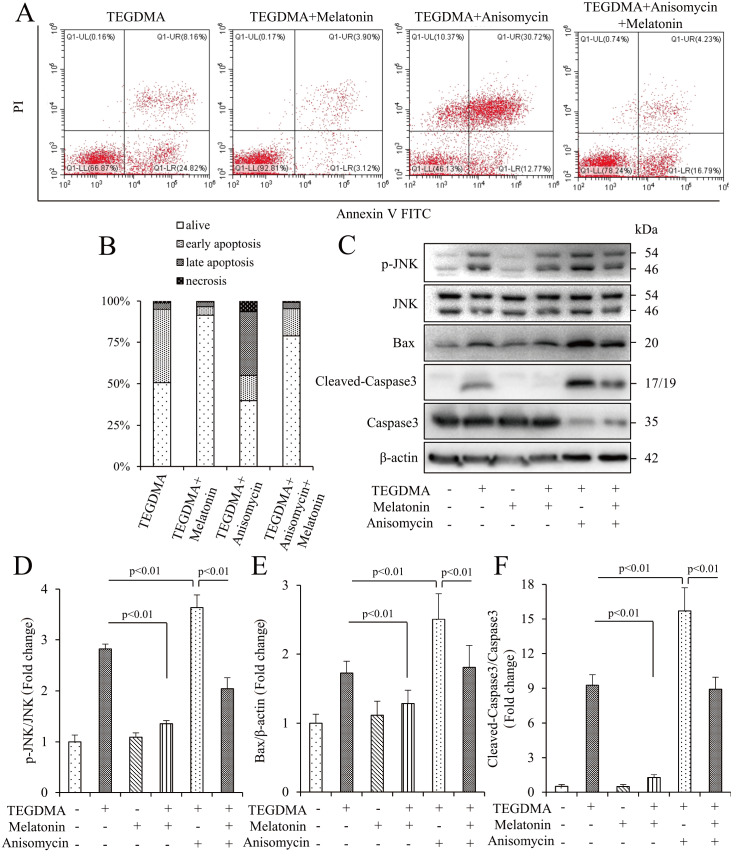
Figure 5 Melatonin prevented TEGDMA-induced apoptosis through the JNK/MAPK pathway (A,B) Flow cytometric analysis after TEGDMA treatment with or without melatonin in the presence of the JNK/MAPK agonist anisomycin. Data are presented as the mean±SD (n=3). (C) Representative western blots in mDPC6T cells with (+) or without (‒) melatonin and anisomycin in the presence of TEGDMA (+) or culture medium (‒). (D) Quantification of protein expression of p-JNK relative to JNK. (E) Quantification of protein expression of Bax relative to β-actin. (F) Quantification of protein expression of cleaved caspase3 relative to caspase3. Data are presented as the mean±SD (n=3).

**Figure 6:**
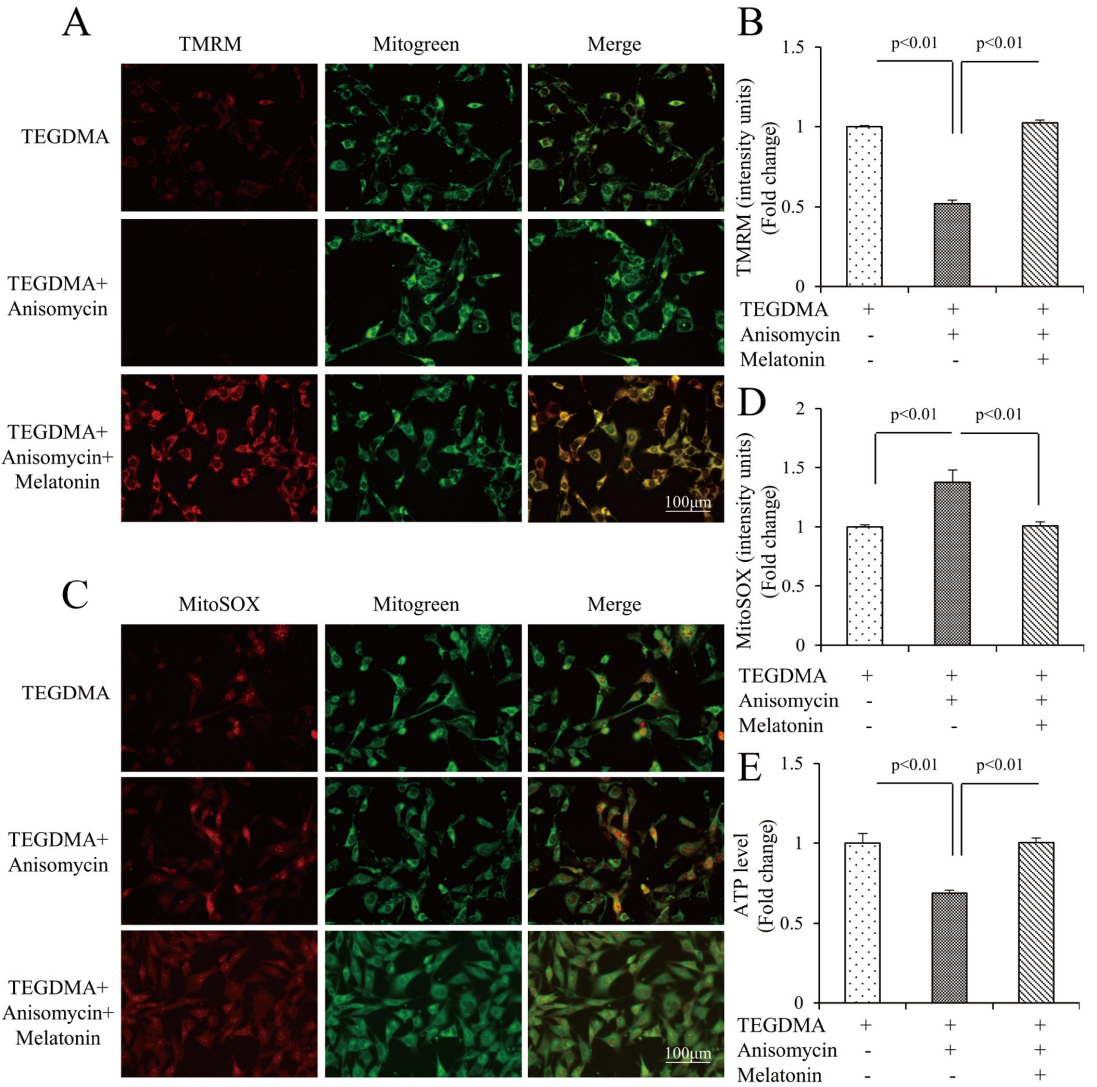
Figure 6 Melatonin attenuated TEGDMA-induced mDPC6T mitochondrial dysfunction through the JNK/MAPK pathway (A,B) Representative images showing TMRM staining (A) and quantification (B) in the indicated groups (n=3). (C,D) Representative images of MitoSOX staining (C) and quantification (D) in the indicated groups (n=3). (E) Cellular ATP levels in the indicated groups (n=3).

We further investigated whether melatonin induces the JNK/MAPK pathway to protect against TEGDMA-induced apoptosis. The JNK agonist anisomycin increased the level of cell apoptosis by approximately 15% compared to TEGDMA alone, while melatonin effectively inhibited this portion of apoptosis, bringing it back to normal level ( Figure 5A,B). Western blot analysis confirmed that melatonin decreased the level of p-JNK and reversed the additional phosphorylation activation of JNK induced by anisomycin ( Figure 5C‒F). Additionally, the results from western blot analysis of apoptosis-related proteins demonstrated that melatonin reversed the increase in cleaved caspase3 and Bax caused by anisomycin ( Figure 5C‒F). Furthermore, the reduced MMP ( Figure 6A,B), elevated mtROS levels ( Figure 6C,D), and decreased ATP production ( Figure 6E) returned to the level of damage induced by TEGDMA with anisomycin following pretreatment with melatonin. This suggests that melatonin alleviates mitochondrial dysfunction and cell apoptosis caused by TEGDMA by inhibiting the JNK/MPK pathway.

## Discussion

Composite restorative materials consist of resin monomers and inorganic fillers, and their polymerization shrinkage can result in the diffusion of resin monomers such as HEMA and TEGDMA through dentin. This process can lead to various harmful effects, including the disruption of normal pulp tissue morphology and physiology, disturbance of cellular redox balance, interference with cell division and genetic material replication, ultimately culminating in pulp cell apoptosis [7]. Melatonin, an endogenously secreted antioxidant and mitochondrial protector, has shown promise in ameliorating mitochondrial dysfunction and inhibiting cell apoptosis. However, the precise protective mechanisms of melatonin against resin monomer damage remain largely unexplored. In our study, we investigated the role of the MAPK signaling pathway, particularly the JNK/MAPK pathway, in TEGDMA-induced mitochondrial apoptosis in preodontoblasts. Our findings highlight the potential therapeutic efficacy of melatonin in managing resin monomer-induced dental pulp injury.

MtROS is a widely accepted initiator of apoptosis through numerous mechanisms, and it is not only related to damage to mitochondrial function but also leads to an apoptotic signaling pathway cascade [ 14, 37, 38]. Notably, as the products of mitochondrial metabolism, mtROS come from the respiratory chain when mitochondria are functionally disordered [15]. It has been found that TEGDMA inhibits complex I in the respiratory chains of mitochondria isolated from guinea pig brain [39]. Nevertheless, our previous research showed that TEGDMA impairs the complex III activity of preodontoblasts, resulting in disordered mitochondria characterized by decreased ATP synthesis and increased production of mtROS [18]. Furthermore, mtROS generation is believed to depend on the membrane potential of mitochondria. Damaged mitochondria, serving as the essential storage pool for the electron chain, promote a reduction in electron flow when the membrane potential across the inner membrane is lost, leading to the production of ROS. As expected, our results demonstrated a significant decrease in MMP and ATP levels, along with an upregulation of mtROS levels, confirming the role of compromised mitochondrial function in TEGDMA-induced apoptosis of mDPC6T cells.

Melatonin, a neurohormone primarily synthesized by the pineal gland, is a multifaceted molecule with diverse physiological functions. Apart from its role in circadian rhythm regulation, melatonin functions as a potent scavenger of mtROS and exhibits significant antioxidant properties. Previous studies have highlighted its established antiapoptotic effects [ 3, 25, 40]. However, studies on the protective role of melatonin on TEGDMA-induced apoptosis of preodontoblasts, as well as its underlying mechanism of action are still lacking. In our investigation, we discovered that pretreatment with melatonin effectively inhibited TEGDMA-induced apoptosis of mDPC6T cells by ameliorating mitochondrial dysfunction. Melatonin exerts its cellular effects through several key mechanisms. Primarily, owing to its highly lipophilic properties, melatonin can permeate cellular and membrane structures, eventually accumulating in the mitochondria [41]. Within the mitochondria, it enhances catalase activity, reduces Ca ^2+^ influx, eliminates residual mtROS, and preserves mitochondrial function [42]. Secondly, melatonin regulates programmed cell death through specific melatonin receptors. The G protein-coupled membrane receptors MT1 and MT2 are recognized as the primary molecules involved in mediating the receptor-dependent pathways of melatonin [ 43, 44]. MT1 and MT2 activate multiple signaling pathways, including the cAMP-response element-binding protein (CREB), phosphatidylinositol 3-kinase (PI3K), and MAPK signaling pathways, integrating various linear inputs to regulate cellular functions such as circadian rhythm, cell differentiation, cumulus expansion, and programmed cell death [ 45‒ 47]. However, further confirmation is required to determine the specific pathway involved in the protective effect of melatonin on preodontoblastic cell apoptosis.

Activated MAPKs exert various biological effects by promoting the phosphorylation of downstream substrates, which then serve as signals in various cell responses, including apoptosis. In our study, the phosphorylation of MAPKs was detected by western blot analysis, and only p-JNK changed with time and concentration when mDPC6T cells were stimulated by TEGDMA. The results are different from those of previous studies [ 24, 25], as they found that the phosphorylation of ERK1/2 and p38 MAPKs was more pronounced. Previous studies examined the effect of TEGDMA on mouse macrophages and treated cells for 24 to 48 h, but we stimulated mDPC6T cells with TEGDMA for approximately 6 h. The distinct cell types and processing conditions may contribute to these inconsistencies. Therefore, we specifically focused on JNK pathway-regulated apoptosis. As expected, pretreatment with SP600125 notably abolished TEGDMA-induced apoptosis, increased mitochondrial function and suppressed mtROS production, and the opposite effect of anisomycin on these results further proved that the JNK pathway is involved in the apoptotic effect of TEGDMA. JNK/MAPK not only is required for the release of cytochrome C from the inner membrane space of mitochondria and the activation of pro-apoptosis proteins, such as Bax and caspase3 [48] but also leads to the inhibition of mitochondrial respiration and electron transport, and damaged mitochondria lead to the release of mtROS and the improvement of MMP [49]. Mechanistically, the direct disruption of the interaction between JNK and mitochondria plays an important role in apoptosis. Krifka *et al*. [4] conducted a comprehensive review of the impact of monomer-induced oxidative stress on central signal transduction pathways, including JNK/MAPK, which reaffirmed the significant involvement of the JNK signaling pathway in TEGDMA-induced cell apoptosis. Nevertheless, the crucial role of JNK necessitates additional validation through overexpression or silencing using siRNA in our future study.

The release of melatonin in response to cellular stress by activating the JNK/MAPK pathway has been reported in various pathological processes [ 31, 45]. In our study, we found that melatonin antagonized mtROS and mDPC6T cell apoptosis caused by TEGDMA or anisomycin alone. Meanwhile, melatonin significantly inhibited cell apoptosis induced by TEGDMA and anisomycin together. Furthermore, melatonin mimicked the effects of the inhibitor SP600125 and abolished the suppressive effects of TEGDMA on p-JNK. Therefore, we propose that melatonin plays a role in mitigating mitochondrial dysfunction-regulated apoptosis partly through the JNK/MAPK pathway. It has been reported that TEGDMA causes mitochondrial oxidative damage via JNK-dependent autophagy to exacerbate mDPC6T cell apoptosis [ 9, 36]. Melatonin can modulate autophagy by various pathways. For example, melatonin and its metabolites adjust various sirtuin pathways related to mitochondrial function and autophagy in the case of stroke [50]. In addition, melatonin-based therapeutics modulated mitophagy in macrophages to ameliorate atherosclerosis [51]. However, whether melatonin eliminates the damaged mitochondria by regulating autophagy in mDPC6T cells has not yet been investigated. The role of the MT1 and MT2 receptors in this context needs further elucidation.

In the present study, we for the first time showcased the remarkable protective effect of melatonin against TEGDMA-induced apoptosis in preodontoblast cells. Nevertheless, certain limitations exist in this study. First, the study exclusively utilized a mouse preodontoblast cell line, warranting the use of primary dental pulp cells to validate the mechanisms underlying melatonin’s protective effect on TEGDMA-induced apoptosis. Moreover, additional *in vivo* investigations are required to verify the preventive effects of TEGDMA on dental pulp injury and confirm the role of the JNK/MAPK signaling pathway.

In summary, the JNK/MAPK signaling pathway appears to be the pivotal mechanism underlying the protective effect of melatonin against TEGDMA-induced mitochondrial apoptosis in mDPC6T cells. As a result, this research potentially contributes to the exploration of melatonin’s application in alleviating TEGDMA-induced dental pulp damage, and provides new perspectives for the development of innovative dental resin materials with enhanced biocompatibility.
