# Corrigendum to: Melatonin protects TEGDMA-induced preodontoblast mitochondrial apoptosis via the JNK/MAPK signaling pathway

**DOI:** 10.3724/abbs.2025031

**Published:** 2025-04-25

**Authors:** Qihao Yu, Ruize Hua, Bingyang Zhao, Dongchao Qiu, Chengfei Zhang, Shengbin Huang, Yihuai Pan

*Acta Biochim Biophys Sin (Shanghai) 2024, 56(3): 393–404*

http://doi: 10.3724/abbs.2023263

In the originally published version of this article, an error was identified in Figure 4A. The corrected figure is provided below. This correction does not materially affect the overall findings and conclusions of the paper. The authors regret this error and apologize for any confusion it may have caused.

**Figure:**
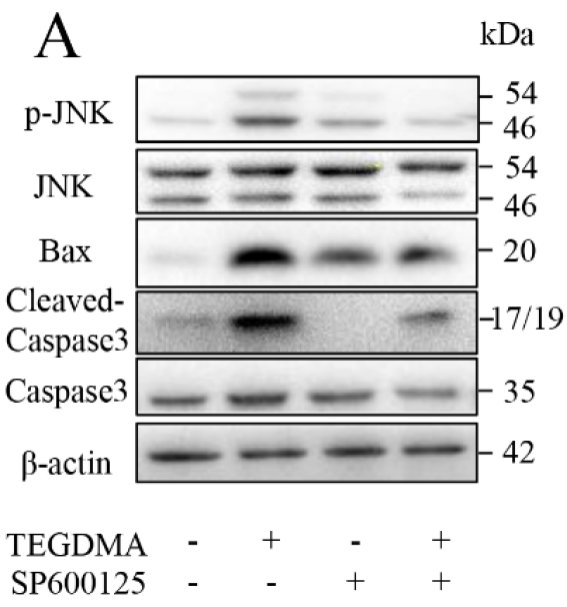
Figure 4A Representative western blots in mDPC6T cells with (+) or without (‒) SP600125 treatment in the presence of TEGDMA (+) or culture medium (‒)

